# Supplementary material for: Caveolin-1 rs1997623 Single Nucleotide Polymorphism Creates a New Binding Site for the Early B-Cell Factor 1 That Instigates Adipose Tissue CAV1 Protein Overexpression
Source: Cells. 2022 Dec 6;11(23):3937. doi: 10.3390/cells11233937 (PMC9738758; doi:10.3390/cells11233937)
Supplement: Supplementary file 1 [file cells-11-03937-s001.zip › cells-1922642-supplementary.pdf]

Caveolin-1 rs1997623 Single Nucleotide Polymorphism Creates a New Binding Site for the Early B-Cell Factor 1 That Instigates Adipose Tissue CAV1 Protein Overexpression

Ashraf Al Madhoun [1,2,*,†], Dania Haddad [1,†], Rasheeba Nizam [1], Lavina Miranda [2], Shihab Kochumon [3], Reeby Thomas [3], Thangavel Alphonse Thanaraj [1], Rasheed Ahmad [3], Milad S. Bitar [1,4] and Fahd Al-Mulla [1,*]

[1] Genetics and Bioinformatics, Dasman Diabetes Institute, Dasman 15462, Kuwait
[2] Animal and Imaging Core Facilities, Dasman Diabetes Institute, Dasman 15462, Kuwait
[3] Immunology & Microbiology Department, Dasman Diabetes Institute, Dasman 15462, Kuwait
[4] Department of Pharmacology and Toxicology, Faculty of Medicine, Kuwait University, Jabriya 46300, Kuwait
* Authors to whom correspondence should be addressed:
ashraf.madhoun@dasmaninstitute.org (A.A.M.); Tel.: +965-2224-2999 (ext. 2805)
fahd.almulla@dasmaninstitute.org (F.A.-M.); Tel.: +965-2224-2999 (ext. 2211)
† These authors contributed equally to this work.

Supplementary Figure S1

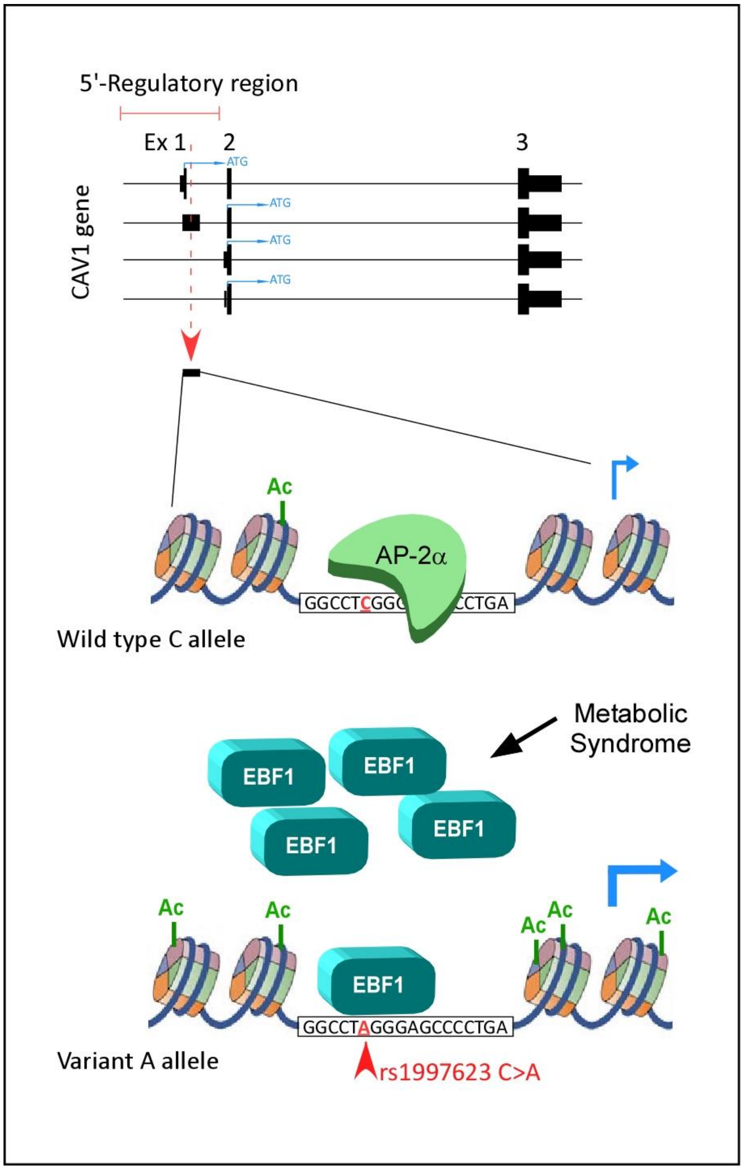

Supplementary Figure S1: A schematic diagram for the human Cav1 gene as described by the UCSC Genome Browser (GRCh37/hg19). The variant rs1997623 is located at 5′ regulatory region within CAV1 gene. However, in one isoform, the variant is located within the non-coding region of the first intron, 214 bp downstream exon 1. Red arrowhead shows rs1997623, blue arrows indicate the translation start sites (ATG, methionine). Sequence analysis revealed the creation of a new binding site for the transcription factor EBF1 instead of AP-2α. **CAV1**, Caveolin-1; **AP-2α,** Activating Enhancer-Binding Protein 2 Alpha; **EBF1**, Early B Cell Factor-1.

Supplementary Figure S2

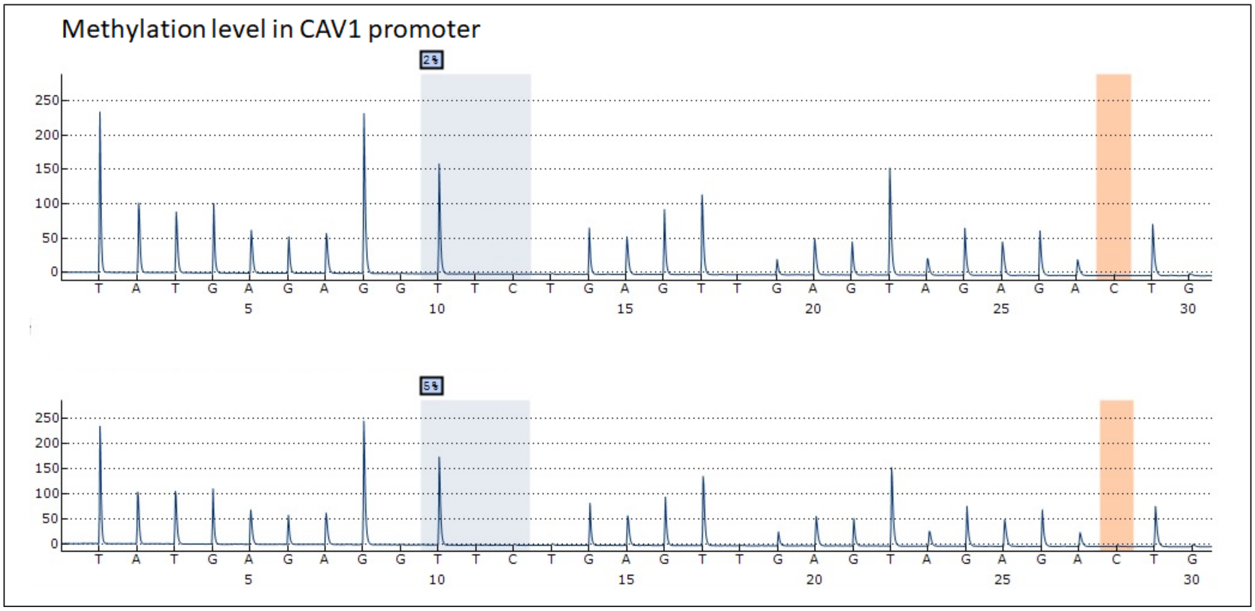

Supplementary Figure S2: DNA methylation analysis by pyrosequencing. Methylation ranged between 2% to 5% across all genotypes CC, CA, and AA.

Supplementary Table S1: List of all human Caveolin-1 transcripts and their corresponding CCDS and proteins.

**Caveolin-1 Transcripts IDs**	**Name**	**bp**	**Protein**	**Translation ID**	**Biotype**	**CCDS**	**UniProt Match**	**Flags**
**ENST00000341049.7**	CAV1-201	2456	178aa	ENSP00000339191.2	Protein coding	CCDS5767	**Q03135-1**	MANE Select, Ensembl Canonical, GENCODE basic, APPRIS P2, TSL:1,
**ENST00000405348.6**	CAV1-205	2652	147aa	ENSP00000384348.1	Protein coding	CCDS55156	**Q03135-2**	GENCODE basic, APPRIS ALT1, TSL:5,
**ENST00000393467.1**	CAV1-202	2628	147aa	ENSP00000377110.1	Protein coding	CCDS55156	Q03135-2	GENCODE basic, APPRIS ALT1, TSL:1,
**ENST00000614113.5**	CAV1-209	1130	115aa	ENSP00000479447.2	Protein coding		**A0A7P0YWJ6**	GENCODE basic, TSL:1,
**ENST00000393468.1**	CAV1-203	845	147aa	ENSP00000377111.1	Protein coding	CCDS55156	Q03135-2	GENCODE basic, APPRIS ALT1, TSL:1,
**ENST00000393470.1**	CAV1-204	598	167aa	ENSP00000377113.1	Protein coding		**E9PCT5**	GENCODE basic, TSL:4,
**ENST00000456473.5**	CAV1-207	557	138aa	ENSP00000389033.1	Protein coding		**C9JKI3**	TSL:4, CDS 3' incomplete,
**ENST00000451122.5**	CAV1-206	1653	86aa	ENSP00000409541.1	Nonsense mediated decay		F8WDM7	TSL:1,
**ENST00000489856.1**	CAV1-208	575	No protein	-	Retained intron		-	TSL:2,

Supplementary Table S2: List of human Caveolin-1 (ENSG00000105974 HGNC: CAV1) transcripts that are affected by rs1997623.

Transcript (strand)	Allele (Tr. allele)	Consequence Type	Position in transcript	Position in CDS	Position in protein	Codons	PolyPhen	CADD
ENST00000451122.5 (+) biotype: nonsense_mediated_decay	G (G)	missense variant NMD transcript variant	298 (out of 1653)	244 (out of 261)	82 (out of 86)	AGG/GGG	unknown(0)	likely benign(2)
ENST00000451122.5 (+) biotype: nonsense_mediated_decay	T (T)	missense variant NMD transcript variant	298 (out of 1653)	244 (out of 261)	82 (out of 86)	AGG/TGG	unknown(0)	likely benign(2)
ENST00000451122.5 (+) biotype: nonsense_mediated_decay	C (C)	synonymous variant NMD transcript variant	298 (out of 1653)	244 (out of 261)	82 (out of 86)	AGG/CGG	-	-
ENST00000341049.7 (+) biotype: protein_coding	C (C)	intron variant	-	-	-	-	-	-
ENST00000341049.7 (+) biotype: protein_coding	G (G)	intron variant	-	-	-	-	-	-
ENST00000341049.7 (+) biotype: protein_coding	T (T)	intron variant	-	-	-	-	-	-
ENST00000393470.1 (+) biotype: protein_coding	C (C)	intron variant	-	-	-	-	-	-
ENST00000393470.1 (+) biotype: protein_coding	G (G)	intron variant	-	-	-	-	-	-
ENST00000393470.1 (+) biotype: protein_coding	T (T)	intron variant	-	-	-	-	-	-
ENST00000489856.1 (+) biotype: retained_intron	C (C)	intron variant non coding transcript variant	-	-	-	-	-	-
ENST00000489856.1 (+) biotype: retained_intron	G (G)	intron variant non coding transcript variant	-	-	-	-	-	-
ENST00000489856.1 (+) biotype: retained_intron	T (T)	intron variant non coding transcript variant	-	-	-	-	-	-
ENST00000614113.5 (+) biotype: protein_coding	C (C)	intron variant	-	-	-	-	-	-
ENST00000614113.5 (+) biotype: protein_coding	G (G)	intron variant	-	-	-	-	-	-
ENST00000614113.5 (+) biotype: protein_coding	T (T)	intron variant	-	-	-	-	-	-
